# *Ulmus davidiana* 60% edible ethanolic extract for prevention of pericyte apoptosis in diabetic retinopathy

**DOI:** 10.3389/fendo.2023.1138676

**Published:** 2023-05-10

**Authors:** Iljin Kim, Jieun Seo, Dong Hyun Lee, Yo-Han Kim, Jun-Hyung Kim, Myung-Bok Wie, Jun-Kyu Byun, Jang-Hyuk Yun

**Affiliations:** ^1^ Department of Pharmacology, Inha University College of Medicine, Incheon, Republic of Korea; ^2^ Faculty of Engineering, Yokohama National University, Yokohama, Japan; ^3^ Department of Ophthalmology, Inha University Hospital, Inha University College of Medicine, Incheon, Republic of Korea; ^4^ College of Veterinary Medicine and Institute of Veterinary Science, Kangwon National University, Chuncheon, Gangwon, Republic of Korea; ^5^ Research Institute of Pharmaceutical Sciences, College of Pharmacy, Kyungpook National University, Daegu, Republic of Korea

**Keywords:** diabetic retinopathy, pericyte apoptosis, endothelial permeability, *Ulmus davidiana*, catechin 7-O-β-D-apiofuranoside

## Abstract

Diabetic retinopathy (DR) is a disease that causes visual deficiency owing to vascular leakage or abnormal angiogenesis. Pericyte apoptosis is considered one of the main causes of vascular leakage in diabetic retina, but there are few known therapeutic agents that prevent it. *Ulmus davidiana* is a safe natural product that has been used in traditional medicine and is attracting attention as a potential treatment for various diseases, but its effect on pericyte loss or vascular leakage in DR is not known at all. In the present study, we investigated on the effects of 60% edible ethanolic extract of *U. davidiana* (U60E) and catechin 7-O-β-D-apiofuranoside (C7A), a compound of *U. davidiana*, on pericyte survival and endothelial permeability. U60E and C7A prevented pericyte apoptosis by inhibiting the activation of p38 and JNK induced by increased glucose and tumor necrosis factor alpha (TNF-α) levels in diabetic retina. Moreover, U60E and C7A reduced endothelial permeability by preventing pericyte apoptosis in co-cultures of pericytes and endothelial cells. These results suggest that U60E and C7A could be a potential therapeutic agent for reducing vascular leakage by preventing pericyte apoptosis in DR.

## 1 Introduction

Diabetic retinopathy (DR) is a disease that causes visual impairment in middle-aged people and is the most common microvascular complication in patients with diabetes (1–3). One of the main causes of DR is an increase in endothelial permeability in the retina, resulting in macular edema, which causes serious visual impairment (2, 4). Pericyte loss is closely related to increased endothelial permeability in DR.

Pericytes surround endothelial cells and play an important role in maintaining the integrity of blood vessels (5, 6). In particular, pericytes interact with endothelial cells to increase the expression of tight junction proteins in endothelial cells, thereby preventing increase in endothelial permeability (7–10). Pericyte loss occurs in the early stages of DR (11–13), therefore, inhibiting pericyte loss may prevent increased endothelial permeability, thereby avoiding serious visual damage such as macular edema. Recently, we confirmed that tumor necrosis factor alpha (TNF-α), which is an important protein that induces pericyte loss *via* apoptosis, is elevated in diabetic retina (8, 14). Additionally, previous studies reveal that high glucose increases apoptosis in retinal pericytes (15); however, few treatments are known to prevent pericyte apoptosis induced by high glucose or TNF-α levels.

*Ulmus davidiana* is a deciduous broad-leaf tree, widely distributed in the east and is a safe natural product used in traditional medicine. *U. davidiana* is known to exhibit pharmacological properties such as antioxidant, anti-inflammatory, anticancer effects (16–18), and its stem or root has long been used for the treatment of various diseases such as edema, mastitis, cancer, inflammation, and rheumatoid arthritis (19–21). Interestingly, *U. davidiana* was known to play a role in preventing apoptosis in various cells such as mouse embryonic fibroblast cells, mouse embryonic liver cells, and rat pheochromocytoma cells [10-12]. However, it is not known how *U. davidiana* extract and compound isolated therefrom affect pericyte apoptosis and endothelial permeability; hence, this was investigated in the present study.

In this study, using a 60% edible ethanolic extract of *U. davidiana* (U60E) and the compound catechin 7-O-β-D-apiofuranoside (C7A) known as the main bioactive component of *U. davidiana* extract (22, 23), the effects and related mechanisms of *U. davidiana* on the increase in pericyte apoptosis and endothelial permeability induced by high glucose and TNF-α were investigated. We demonstrated that U60E and C7A prevent pericyte apoptosis by blocking the activities of p38 and JNK, which are increased by high glucose and TNF-α levels. Additionally, U60E and C7A restored the decreased ZO-1 expression and increased permeability in endothelial cells caused by pericyte apoptosis induced by high glucose and TNF-α when pericytes and endothelial cells were co-cultured. Taken together, these results suggest a potential therapeutic benefit of U60E and C7A in preventing pericyte apoptosis in DR.

## 2 Materials and methods

### 2.1 Cell cultures

Human placental pericytes (PromoCell, Heidelberg, Germany) and human retinal microvascular endothelial cells (HRMECs; ACBRI, Kirkland, WA, USA) were maintained in pericytes medium including growth factors (PromoCell) and M199 medium (HyClone, Logan, UT, USA) with 20% fetal bovine serum (FBS), respectively. In an incubator with a humidified environment containing 5% CO_2,_ the cells were cultured at 37°C.

### 2.2 Reagents and antibodies

R&D Systems (Minneapolis, MA, USA) provided the recombinant human TNF-α, whereas Millipore (St. Louis, MO, USA) provided the p38 and JNK activator anisomycin, p38 inhibitor SB203580, JNK inhibitor SP600125, glucose, and mannitol, respectively. Cell Signaling Technology (Danvers, MA, USA) provided the primary anti-phospho-p38, anti-p38, anti-phospho-JNK, anti-JNK, anti-phospho-Erk1/2, anti-Erk1/2, and anti-cleaved caspase-3 antibodies. Thermo Fisher Scientific (Waltham, MA) provided the anti-ZO-1 and anti-occludin antibodies. Santa Cruz Biotechnology (Dallas, TX, USA) provided the anti-β-tubulin and peroxidase-conjugated secondary antibodies. ChemFaces (Wuhan, Hubei, China) provided the C7A.

### 2.3 Preparation of U60E extracts

*U. davidiana* (branches with barks) was officially collected in June 2020 in Dolsan-eup, Yeosu-si, Jeollanam-do, Republic of Korea. A voucher specimen (UDB2020-06) was placed in the herbarium of the Kangwon National University College of Forest & Environmental Sciences. *U. davidiana* was used as experimental materials by removing impurities, washing, and shading. Once, 10 kg of *U. davidiana* were extracted at room temperature using 60% edible ethanol. After that, the extract was concentrated by eliminating 60% edible ethanol while under vacuum, producing 570 g (U60E) (**Supplementary Figure 1**). Dimethyl sulfoxide (DMSO) was used to dissolved the dried 60% edible ethanol extract before it was diluted in a cell culture medium.

### 2.4 Cell viability assay

The 3-(4,5-di methylthiazol-2-yl)-2,5-diphenyltetrazolium bromide (MTT) labeling kit (Millipore Sigma) was used to assess the cell viability. Briefly, U60E and/or TNF-α were applied to 5 × 10^3^ cells planted into 96-well plates for 72 h. After 3 h, the cells were treated with MTT reagent (5 mg/mL), and the formazan product produced was assessed by measuring the intensity of the absorbance at 570 nm.

### 2.5 FACS analysis

5 × 10^5^ cells were exposed to the indicated reagents for 72 h in order to identify apoptosis. The cells were harvested and given two PBS washes. After that, the cells were labelled with FITC Annexin V and PI (BD Biosciences, Franklin Lakes, NJ, USA) for 15 min, and staining was measured by flow cytometry on a FACSCalibur (BD Biosciences, Franklin Lakes, NJ, USA). Data were examined using the FlowJo program. Cells that were positive for Annexin V were considered to be apoptotic.

### 2.6 Western blot analysis

A solution comprising 20 mM Tris (pH 7.5), 150 mM sodium chloride (NaCl), 1% Triton X-100, and a cocktail of protease inhibitors was used to lyse the cells. Proteins from cell lysates were put into nitrocellulose membranes after being electrophoretically separated on 7–10% sodium dodecyl sulfate-polyacrylamide gel electrophoresis gels. The blots were incubated with the indicated primary antibodies (1:1000) at 4°C overnight, and then probed with secondary antibodies (1:5000) at room temperature for 1 h. The blots were subsequently exposed to a film after being treated with an enhanced chemiluminescence substrate (Thermo Fisher Scientific).

### 2.7 Bromodeoxyuridine enzyme-linked immunosorbent assay for cell proliferation estimation

The manufacturer’s instructions were followed while measuring cell proliferation with the Cell Proliferation BrdU ELISA kit (Roche Diagnostics, Indianapolis, IN, USA). Cells treated with the indicated reagents for 72 h were labelled with 10 μM BrdU for 1 h and then incubated with an anti-BrdU peroxidase-conjugated antibody for 90 min. After washing, the substrate reaction, which was gauged using an ELISA plate reader at 450 nm, was used to identify the bound peroxidase.

### 2.8 Endothelial permeability assay

By measuring the flux of Evans blue (MilliporeSigma)-labeled bovine serum albumin (BSA; MilliporeSigma) through the cell monolayers using a Transwell plate, endothelial permeability was determined (8). Briefly, HRMECs or pericytes were plated on the top or bottom sides of the Transwell filter (Costar, Washington, DC, USA) and cultured in normal or high glucose conditions with U60E and/or TNF-α for an additional 72 h on each side. Evans blue dye was used in the culture medium to measure endothelial permeability. The optical properties of the medium in the bottom chamber were assessed spectrophotometrically at 650 nm (Tecan Infinite M200PRO).

### 2.9 Statistics analysis

A standard two-tailed Student’s *t-*test was used to conduct statistical analyses, and statistical significance was set at p < 0.05. Mean ± standard deviation (SD) was used to present quantitative data and figures.

## 3 Results

### 3.1 U60E and C7A prevent decrease in cell viability in the pericytes

**Figure 1:**
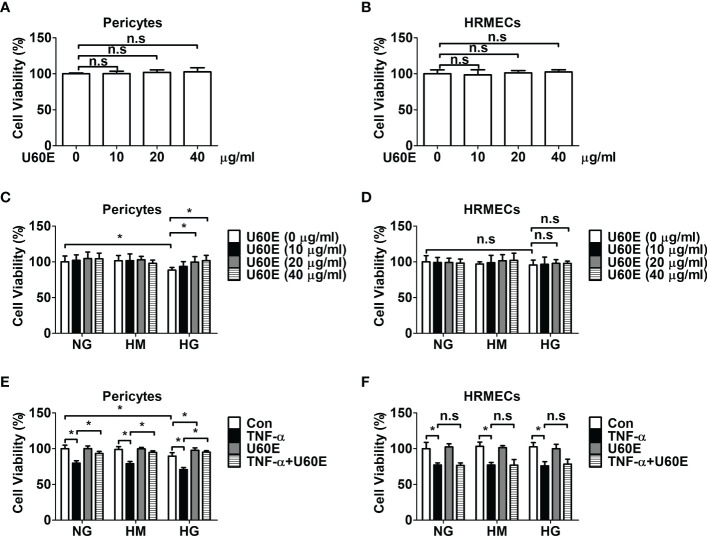
Effect of U60E on cell viability of pericytes and human retinal microvascular endothelial cells (HRMECs). Pericytes **(A)** and HRMECs **(B)** were treated with U60E for 72 h at indicated doses. The cell viability was determined by the MTT assay. The bar graph represents the means ± standard deviation (SD) (*n* = 5). Pericytes **(C)** and HRMECs **(D)** were treated with U60E for 72 h at indicated doses under the conditions of high glucose (HG; 30 mM glucose). Normal glucose (NG; 5 mM glucose) and high mannitol (HM; 5 mM glucose and 25 mM mannitol) were used as controls. The cell viability was determined by the MTT assay. The bar graph represents the means ± SD (*n* = 5). *P < 0.05. Pericytes **(E)** and HRMECs **(F)** were treated with U60E (20 μg/ml) for 72 h under the conditions of HG, with or without tumor necrosis factor α (TNF-α) (100 ng/ml). The cell viability was determined by the MTT assay. The bar graph represents the means ± SD (*n* = 5). No significance (n.s.) indicates *P* > 0.05, *P < 0.05.

The effect of U60E on the viability of pericytes and endothelial cells was investigated using an MTT assay. Treatment with U60E alone did not affect the viability of pericytes and HRMECs at any of the indicated concentrations (**Figures 1A, B**). However, when exposed to high levels of glucose or TNF-α, U60E prevented the decrease in cell viability among pericytes, but not in HRMECs (**Figures 1C–F**). Next, we tried to find out which compound in U60E plays this role. In a previous study, we confirmed that C7A (**Supplementary Figure 2**) is an important bioactive compound related to cell survival isolated from *U. davidiana* extract, and U60E also contains abundant C7A(24). Therefore, we hypothesized that C7A contained in U60E is an important compound in preventing the decrease in cell viability of pericytes. Like U60E, when exposed to high levels of glucose or TNF-α, C7A prevented the decrease in cell viability among pericytes, but not in HRMECs (**Supplementary Figures 3A–D**). These results indicate that C7A contained in U60E prevents the decrease in pericyte cell viability in DR.

### 3.2 U60E and C7A prevent pericyte apoptosis

**Figure 2:**
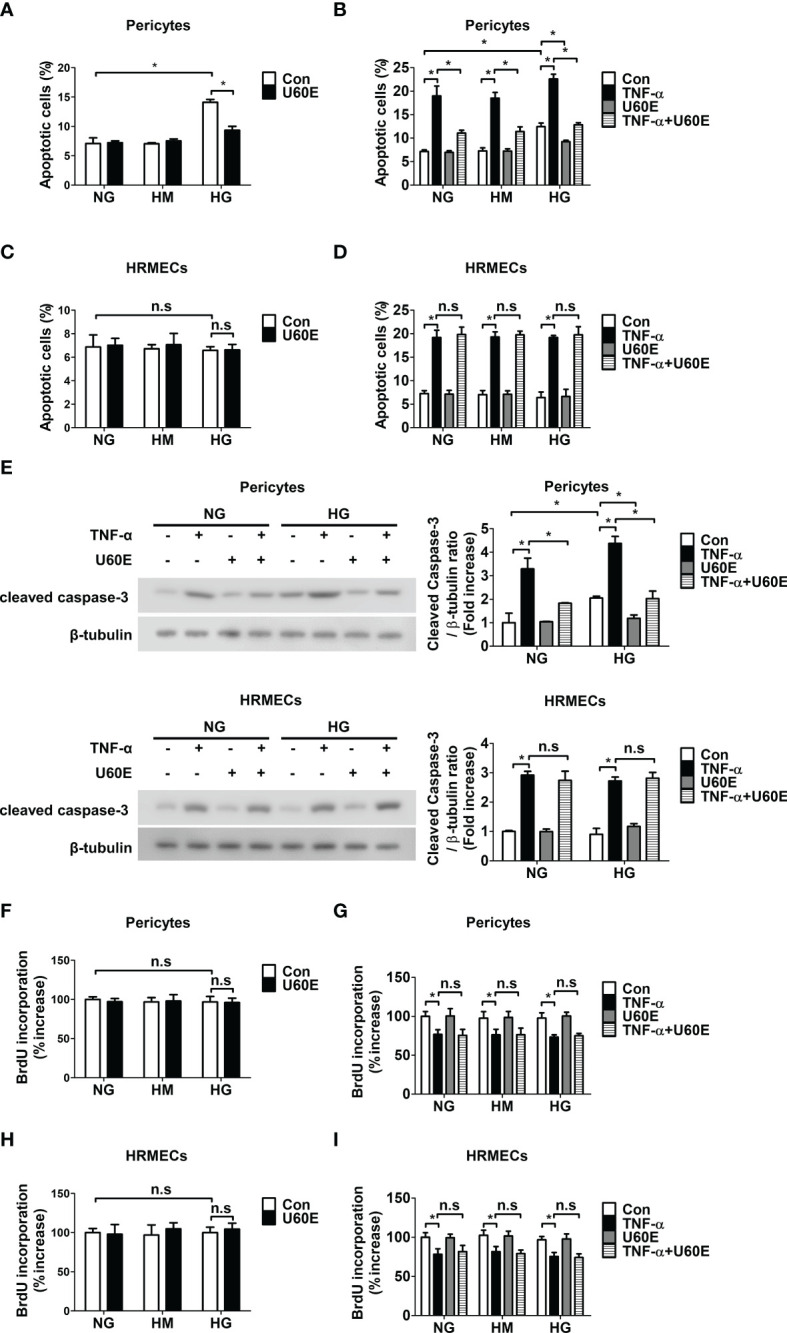
Effect of U60E on survival and proliferation in pericytes and human retinal microvascular endothelial cells (HRMECs). **(A-I)** Pericytes and HRMECs were treated with U60E (20 μg/ml) for 72 h under the conditions of high glucose (HG; 30 mM glucose), with or without tumor necrosis factor α (TNF-α) (100 ng/ml). Normal glucose (NG; 5 mM glucose) and high mannitol (HM; 5 mM glucose and 25 mM mannitol) were used as controls. Cell apoptosis of pericytes **(A, B)** and HRMECs **(C, D)** was determined by Annexin V/PI staining and flow cytometric analysis. The apoptotic cells were expressed as a percentage of apoptotic cells in the total cell population. The bar graph represents the means ± standard deviation (SD) (*n* = 3). **(E)** The cleaved caspase-3 expression was determined by western blot analysis. β-tubulin were used as controls. The right histogram showed quantitative densitometric analysis. The bar graph represents the means ± standard deviation (SD) (*n* = 3). Cell proliferation of pericytes **(F, G)** and HRMECs **(H, I)** was determined by 5’-bromodeoxy-2’-uridine (BrdU) proliferation ELISA. The bar graph represents the means ± SD (*n* = 5). No significance (n.s.) indicates *P* > 0.05, *P < 0.05.

Cell apoptosis and proliferation were measured using annexin-V/PI flow cytometric analysis, western blot analysis, and BrdU proliferation ELISA assays, to determine how U60E prevents the decrease in pericyte cell viability in DR. Interestingly, U60E effectively prevented pericyte apoptosis induced by high glucose and TNF-α (**Figures 2A, B**), however, there was no effect on HRMEC apoptosis (**Figures 2C, D**). U60E also effectively prevented increased level of apoptosis-associated protein (cleaved caspase-3) by high glucose and TNF-α in pericytes, but had no effect on HRMECs (**Figure 2E**). In addition, U60E did not affect the proliferation of pericytes or HRMECs exposed to high glucose and TNF-α (**Figures 2F–I**). C7A also effectively prevented pericyte apoptosis induced by high glucose and TNF-α (**Supplementary Figure 4A**), but had no effect on HRMEC apoptosis (**Supplementary Figure 4B**). Additionally, C7A effectively prevented high glucose- and TNF-α-induced increased expression of cleaved caspase-3 in pericytes, but did not affect HRMECs (**Supplementary Figure 4C**). C7A also did not affect the proliferation of pericytes or HRMECs exposed to high glucose and TNF-α (**Supplementary Figures 4D, E**). These results indicated that C7A contained in U60E prevents pericyte apoptosis in DR.

### 3.3 U60E and C7A prevent pericyte apoptosis by reducing the increase in p38 and JNK activity in the diabetic retina

**Figure 3:**
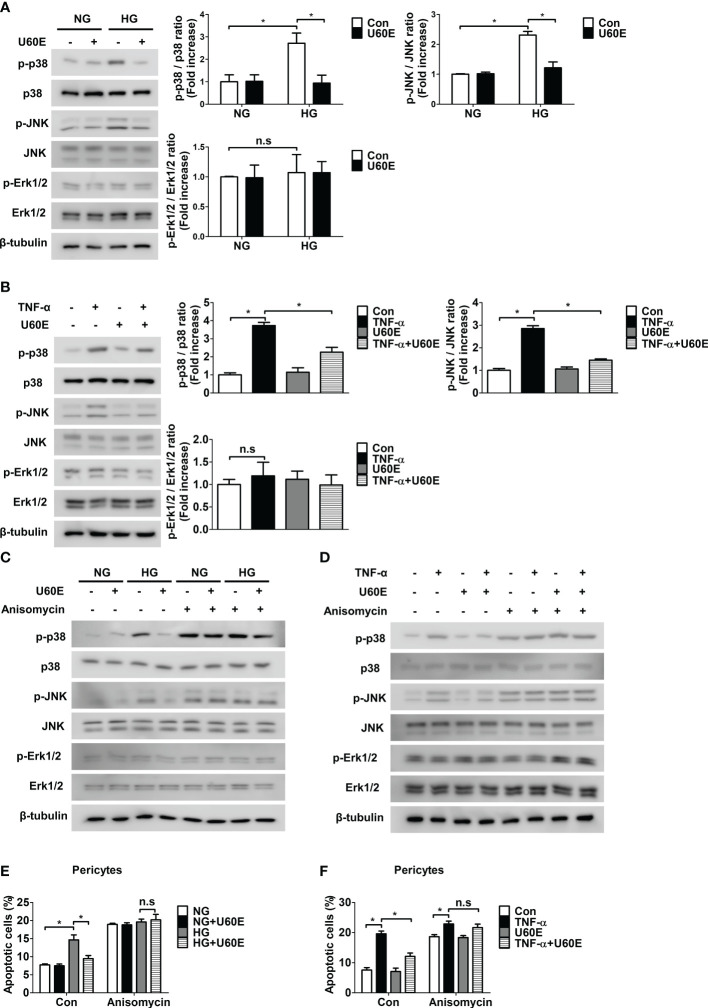
Involvement of p38 and JNK signaling in U60E-induced pericyte survival. **(A–D)** Pericytes were treated with U60E (20 μg/mL), tumor necrosis factor α (TNF-α) (100 ng/ml), and/or anisomycin (100 ng/ml) for 30 min under conditions exposed to normal glucose (NG; 5 mM glucose) or high glucose (HG, 30 mM glucose) for 24 h. The phosphorylation of p38 (p-p38), JNK (p-JNK), and Erk1/2 (p-Erk1/2) was determined by western blot analysis. p38, JNK, Erk1/2, and β-tubulin were used as controls. **(A, B)** The right histogram showed quantitative densitometric analysis. The bar graph represents the means ± standard deviation (SD) (*n* = 3). **(E, F)** Pericytes were treated with U60E (20 μg/mL), TNF-α (100 ng/ml), and/or anisomycin (100 ng/ml) for 72 h under NG or HG conditions. Cell apoptosis was determined by Annexin V/PI staining and flow cytometric analysis. The apoptotic cells were expressed as a percentage of apoptotic cells in the total cell population. The bar graph represents the means ± SD (*n* = 3). No significance (n.s.) indicates *P* > 0.05, *P < 0.05.

The mechanism by which U60E and C7A prevent pericyte apoptosis was determined. High glucose and TNF-α levels increased the phosphorylation of p38 and JNK, but not Erk1/2, in a time-dependent manner in pericytes (**Supplementary Figures 5A, B**). However, U60E played a role in reducing the phosphorylation of p38 and JNK, which was increased by high glucose and TNF-α levels (**Figures 3A, B**). To further evaluate the role of p38 and JNK in pericyte apoptosis, we used the p38 inhibitor SB203580, JNK inhibitor SP600125, and p38 and JNK activator anisomycin. SB203580 completely blocked p38 phosphorylation, but not JNK, while SP600125 completely blocked JNK phosphorylation, but not p38 (**Supplementary Figures 6A, B**). However, both SB203580 and SP600125 played a role in reducing pericyte apoptosis induced by high glucose and TNF-α levels (**Supplementary Figures 6C, D**). In addition, anisomycin completely prevented the inhibition of high glucose- and TNF-α-induced phosphorylation of p38 and JNK and apoptosis by U60E in pericytes (**Figures 3C–F**). Like U60E, C7A also played a role in reducing the phosphorylation of p38 and JNK, which was increased by high glucose and TNF-α levels (**Supplementary Figures 7A, B**). In addition, anisomycin also completely prevented the inhibition of high glucose- and TNF-α-induced phosphorylation of p38 and JNK and apoptosis by C7A in pericytes (**Supplementary Figures 7A–D**). These results indicated that C7A contained in U60E inhibited apoptosis by blocking the activation of p38 and JNK in the pericytes of DR.

### 3.4 U60E and C7A prevent endothelial permeability by blocking pericyte apoptosis

**Figure 4:**
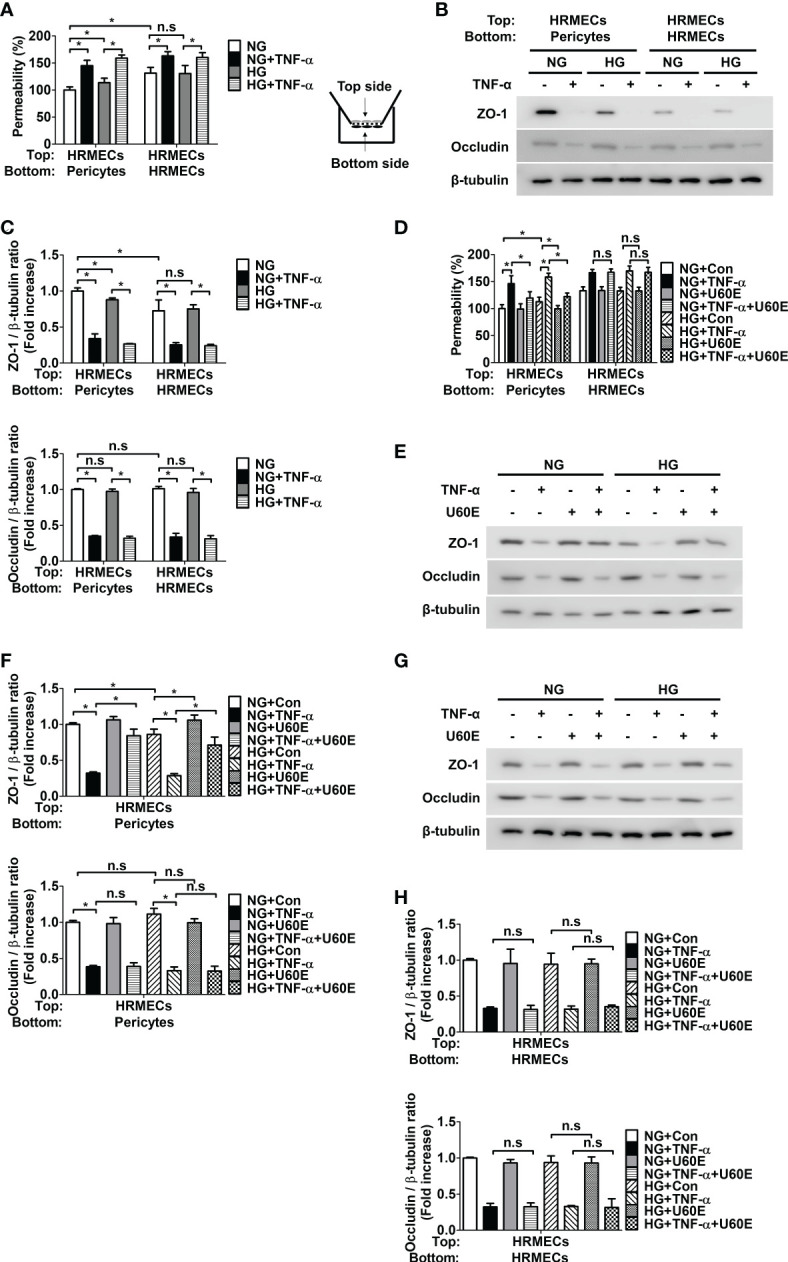
Effect of U60E on the *in vitro* permeability in co-cultures of pericytes and human retinal microvascular endothelial cells (HRMECs) and the tight junction protein expression in HRMECs. **(A)** Pericytes and HRMECs incubated on the indicated side of the Transwells as depicted at right. Pericytes and HRMECs were treated with tumor necrosis factor α (TNF-α) (100 ng/ml) under normal glucose (NG; 5 mM glucose) or high glucose (HG, 30 mM glucose) for 72 h. The permeability was measured using Evans blue dye (*n* = 5). **(B)** The tight junction protein expression of ZO-1 and occludin was measured from the top side HRMECs lysates obtained by **(A)**. **(C)** Quantitative densitometric analysis in **(B)** to calculate the ratio of each protein to β-tubulin. The bar graph represents the means ± standard deviation (SD) (*n* = 3). **(D)** Pericytes and HRMECs were incubated on the indicated side of the Transwells and then treated with U60E (20 μg/mL) and/or TNF-α under conditions exposed to NG or HG for 72 h. The permeability was measured using Evans blue dye (*n* = 5). **(E–H)** The tight junction protein expression of ZO-1 and occludin was measured from the top side HRMECs lysates under conditions for co-culture of pericytes and HRMECs **(E, F)** or conditions for culturing only HRMECs **(G, H)** obtained by **(D)**. Quantitative densitometric analysis was performed to calculate the ratio of each protein to β-tubulin **(F, H)**. The bar graph represents the means ± SD (*n* = 3). No significance (n.s.) indicates *P* > 0.05, *P < 0.05.

Since it was previously confirmed that pericyte survival prevents endothelial permeability by increasing the tight junction protein ZO-1, but not occludin (8), we hypothesized that U60E and C7A would also prevent endothelial permeability by increasing the tight junction protein ZO-1. When pericytes and HRMECs were co-cultured on both sides of the Transwell membrane, the permeability was lower than that of HRMECs cultured on both sides of the Transwell (**Figure 4A**). In addition, when pericytes and HRMECs were co-cultured, endothelial permeability was increased in both high glucose and TNF-α, conditions that induce pericyte apoptosis (**Figure 4A**). However, when only HRMECs were co-cultured on both sides of the Transwell, endothelial permeability increased only when treated with TNF-α and not high glucose (**Figure 4A**). Similarly, when pericytes and HRMECs were co-cultured, the expression level of ZO-1 protein in HRMECs on the top side of the Transwell was higher than that in HRMECs cultured alone, but there was no change in occludin protein levels (**Figures 4B, C**). In addition, when pericytes and HRMECs were co-cultured, both high glucose and TNF-α, decreased the expression level of ZO-1 (**Figures 4B, C**). However, when only HRMECs were co-cultured on both sides of the Transwell, the expression level of ZO-1 decreased only when treated with TNF-α and not high glucose (**Figures 4B, C**). Furthermore, when pericytes and HRMECs were co-cultured as well as when only HRMECs were cultured, the expression level of occludin was decreased only when treated with TNF-α, not high glucose (**Figures 4B, C**). These results indicate that pericyte apoptosis induced by high glucose and TNF-α increases endothelial permeability by reducing the expression of ZO-1 in endothelial cells, and TNF-α not only induces pericyte apoptosis, but also increases endothelial permeability by directly decreasing the expression of ZO-1 and occludin in endothelial cells. On the other hand, high glucose did not directly affect permeability or tight junction protein levels in endothelial cells. Next, we investigated the effect of U60E and C7A on endothelial permeability. U60E prevented endothelial permeability only when pericytes and HRMECs were co-cultured, not when only HRMECs were cultured (**Figure 4D**). Similarly, U60E restored the ZO-1 expression level decreased by high glucose or TNF-α only when pericytes and HRMECs were co-cultured (**Figures 4E, F**), but not when only HRMECs were cultured (**Figures 4G, H**). In addition, U60E did not affect occludin expression levels when co-cultured with pericytes and HRMECs, or when only HRMECs were cultured (**Figures 4E–H**). Like U60E, C7A also prevented endothelial permeability only when pericytes and HRMECs were co-cultured, not when only HRMECs were cultured (**Supplementary Figure 8A**). C7A also restored the ZO-1 expression level decreased by high glucose or TNF-α only when pericytes and HRMECs were co-cultured (**Supplementary Figures 8B, C**), but not when only HRMECs were cultured (**Supplementary Figures 8D, E**). In addition, C7A also did not affect occludin expression levels when co-cultured with pericytes and HRMECs, or when only HRMECs were cultured (**Supplementary Figures 8B–E**). These results suggest that C7A contained in U60E is not directly involved in permeability of endothelial cells, but prevents pericyte apoptosis induced by high glucose and TNF-α levels, thereby preventing an increase in endothelial permeability.

## 4 Discussion

In this study, we investigated the effect of U60E on endothelial permeability in DR. In our previous studies, we confirmed that U60E prevents VEGF-induced endothelial cell proliferation, tube formation, and migration; therefore, we hypothesized that U60E could be an effective treatment for retinopathy caused by abnormal angiogenesis, such as proliferative DR (25). However, the effect of U60E on endothelial permeability, another major cause of DR, is unknown. In the present study, U60E did not directly affect endothelial permeability when HRMECs were cultured alone (**Figure 4D**), but we confirmed that U60E prevented the increase in endothelial permeability induced by high glucose and TNF-α when HRMECs and pericytes were co-cultured (**Figure 4D**). Therefore, U60E may prevent endothelial permeability through pericytes.

Pericyte loss occurs in the early stage of DR (11–13) and is closely related to an increase in endothelial permeability (8, 10). According to our previous study, we confirmed that increased TNF-α in the diabetic retina induces pericyte apoptosis, leading to pericyte loss, thereby reducing ZO-1 expression in endothelial cells and increasing endothelial permeability (14). In addition, high glucose levels are known to induce rat retinal pericyte apoptosis (15), and we previously confirmed that high glucose induces pericyte apoptosis (26). *U. davidiana* extract also prevents apoptosis in various cells, such as mouse embryonic fibroblast cells, mouse embryonic liver cells, and rat pheochromocytoma cells [10–12]. In addition, in a previous study, we confirmed that the C7A compound contained in *U. davidiana* extract had bioactivity related to cell survival, and U60E also contained C7A (24). In addition, C7A is known as a representative bioactive compound of *U. davidiana* extract (22, 23). Therefore, we hypothesized that C7A contained in *U. davidiana* extract could prevent endothelial permeability by preventing pericyte apoptosis induced by high glucose or TNF-α in DR. In the present study, we confirmed that U60E and C7A prevent pericyte apoptosis induced by high glucose and TNF-α (**Figures 2A–E**, **Supplementary Figures 4A–C**). In addition, when HRMECs and pericytes were co-cultured, we confirmed that U60E and C7A restored the decrease in the expression of ZO-1 and the increase in permeability induced by high glucose and TNF-α in endothelial cells (**Figures 4D–F**, **Supplementary Figures 8A–C**). These results suggest that C7A contained in U60E prevents endothelial permeability by inhibiting pericyte apoptosis induced by high glucose and TNF-α levels.

DR is the most common microvascular complication in diabetic patients and is the leading cause of blindness between the ages of 20 and 64 (27). The two main causes of DR are retinal vascular leakage and abnormal retinal angiogenesis (27). Interestingly, through previous study (25) and this study, almost all *in vitro* experiments confirmed that U60E or C7A prevent both retinal vascular leakage and retinal angiogenesis in the condition of DR. These are interesting results that U60E or C7A can block both major causes of DR. However, in the case of our studies, there is a limitation that only *in vitro* experiments were conducted. Through *in vivo* experiments, it seems necessary to confirm whether U60E and C7A actually prevent retinal vascular leakage and abnormal retinal angiogenesis in DR. Furthermore, it seems necessary to confirm whether U60E and C7A are actually effective in DR patients through clinical trials. In addition, although U60E and C7A are extract and compound of the *U. davidiana*, which is safe natural product used in traditional medicine, respectively, additional confirmation of the side effects of U60E or C7A seems necessary to safely use the agents in clinical practice. Although more research is needed for these agents to be commercialized in clinical practice, the discovery of potential disease treatment agents at the cell level, such as this study, is thought to be a basic stepping stone for the development of actual clinical treatments in the future.

In this study, we investigated the mechanisms by which U60E and C7A prevent pericyte apoptosis induced by high glucose and TNF-α levels. Previously, we confirmed that high glucose and TNF-α induce pericyte apoptosis by decreasing the expression level of Bcl-2, a pro-survival protein, and increasing the expression level of Bax, a pro-apoptotic protein (14). However, the mechanism by which high glucose and TNF-α induce pericyte apoptosis is unclear. TNF-α and high levels of glucose have been shown to increase the activation of p38, JNK, and ERK1/2 in various cells (28–32). It is also well known that p38, JNK, and ERK1/2 activation is closely related to the apoptotic pathway (33). Recent studies revealed a component of *U. davidiana* extract was known to inhibit TNF-α-induced activation of p38, JNK, and ERK1/2 in human dermal fibroblasts (34). Therefore, we hypothesized that U60E and C7A might prevent pericyte apoptosis by blocking the activation of p38, JNK, or ERK1/2. We confirmed that high glucose and TNF-α induce pericyte apoptosis by activating p38 and JNK (**Supplementary Figures 6A–D**). However, high levels of glucose and TNF-α did not activate ERK1/2 in the pericytes (**Supplementary Figures 5A, B**). In addition, we confirmed that U60E and C7A prevented pericyte apoptosis by blocking the activation of p38 and JNK, but were not involved in ERK1/2 (**Figures 3C–F**, **Supplementary Figures 7A–D**). These results suggest that C7A contained in U60E prevents apoptosis by blocking the activation of p38 and JNK in pericytes caused by high glucose and TNF-α in DR.

## 5 Conclusions

DR is the leading cause of vision damage in working-aged people and is currently the most common microvascular complication despite treating DR through glycemic control and photocoagulation. One of the main causes of DR is vascular leakage in the retina, and when vascular leakage occurs in the retina, it causes serious vision damage. Pericytes play a role in interacting with endothelial cells to increase the tight junction protein ZO-1 of the endothelial cells, thereby reducing endothelial permeability. Therefore, when pericyte loss occurs, vascular leakage is more likely to occur. Interestingly, pericyte loss is one of the most characteristic and earliest changes in DR. In addition, pericyte apoptosis occurs in DR, resulting in pericyte loss. Therefore, if a substance capable of preventing apoptosis of pericytes is found, it may be used as a therapeutic agent for DR by preventing retinal vascular leakage. *U. davidiana* is a safe natural product that has been used in traditional medicine and is attracting attention as a potential treatment for various diseases, but its effect on pericyte loss or vascular leakage in DR is not known at all.

In this study, we confirmed that C7A, a major compound of *U. davidiana* and U60E, prevented the reduction of pericyte cell viability in DR. In addition, U60E and C7A prevented pericyte apoptosis by blocking the activity of p38 and JNK induced by high glucose and TNF-α in DR. U60E and C7A also prevented the increase in endothelial permeability caused by pericyte apoptosis in DR. These results suggest that U60E and C7A may be a potential therapeutic agents in DR by preventing pericyte apoptosis.

## Data availability statement

The original contributions presented in the study are included in the article/**Supplementary Material**, further inquiries can be directed to the corresponding authors.

## Author contributions

IK, JS, and DL wrote the manuscript and performed experiments. Y-HK, J-HK, and M-BW analyzed the data and reviewed the manuscript. J-HY and J-KB designed the study and wrote/edited the manuscript. All authors have read and agreed to the published version of the manuscript.
