# Supplementary material for: Ulmus davidiana 60% edible ethanolic extract for prevention of pericyte apoptosis in diabetic retinopathy
Source: Front Endocrinol (Lausanne). 2023 May 10;14:1138676. doi: 10.3389/fendo.2023.1138676 (PMC10206296; doi:10.3389/fendo.2023.1138676)
Supplement: Supplementary file 1 [file DataSheet_1.docx]

Supplementary Material

*Ulmus davidiana* 60% edible ethanolic extract for prevention of pericyte apoptosis in diabetic retinopathy

**Iljin Kim^1,#^, Jieun Seo^2,#^, Dong Hyun Lee^3,#^, Yo-Han Kim^4^, Jun-Hyung Kim^4^, Myung-Bok Wie^4^, Jun-Kyu Byun^5,*^, Jang-Hyuk Yun^4, *^**

^1^Department of Pharmacology, Inha University College of Medicine, Incheon, Republic of Korea.

^2^Faculty of Engineering, Yokohama National University, Yokohama, 240-8501, Japan.

^3^Department of Ophthalmology, Inha University Hospital, Inha University College of Medicine, Incheon, Republic of Korea.

^4^College of Veterinary Medicine and Institute of Veterinary Science, Kangwon National University, Chuncheon, Gangwon, 24341, Republic of Korea.

^5^Research Institute of Pharmaceutical Sciences, College of Pharmacy, Kyungpook National University, Daegu 41566, Republic of Korea.

^#^ These authors contributed equally to this work.

*** Correspondence:**

**Jun-Kyu Byun, D.V.M., Ph.D.**, Research Institute of Pharmaceutical Sciences, College of Pharmacy, Kyungpook National University, Daegu 41566, Republic of Korea. E-mail: jkbyun@knu.ac.kr Phone: 82-53-950-8585 Fax: 82-53-950-8557

**Jang-Hyuk Yun, D.V.M., Ph.D.**, College of Veterinary Medicine and Institute of Veterinary Science, Kangwon National University, Chuncheon, Gangwon, 24341, Republic of Korea. E-mail: yunjh@kangwon.ac.kr Phone: 82-33-250-8653 Fax: 82-33-259-5625

# Supplementary Figures

**
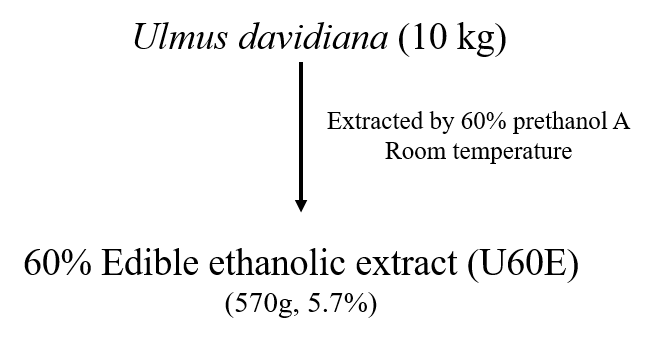
**

**Supplementary Figure 1.** Scheme of extraction of U60E.

**
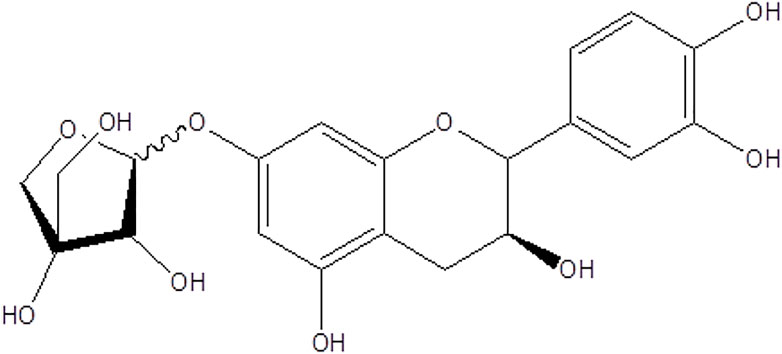
**

**Supplementary Figure 2.** Chemical structure of C7A isolated from *U. davidiana.*

**
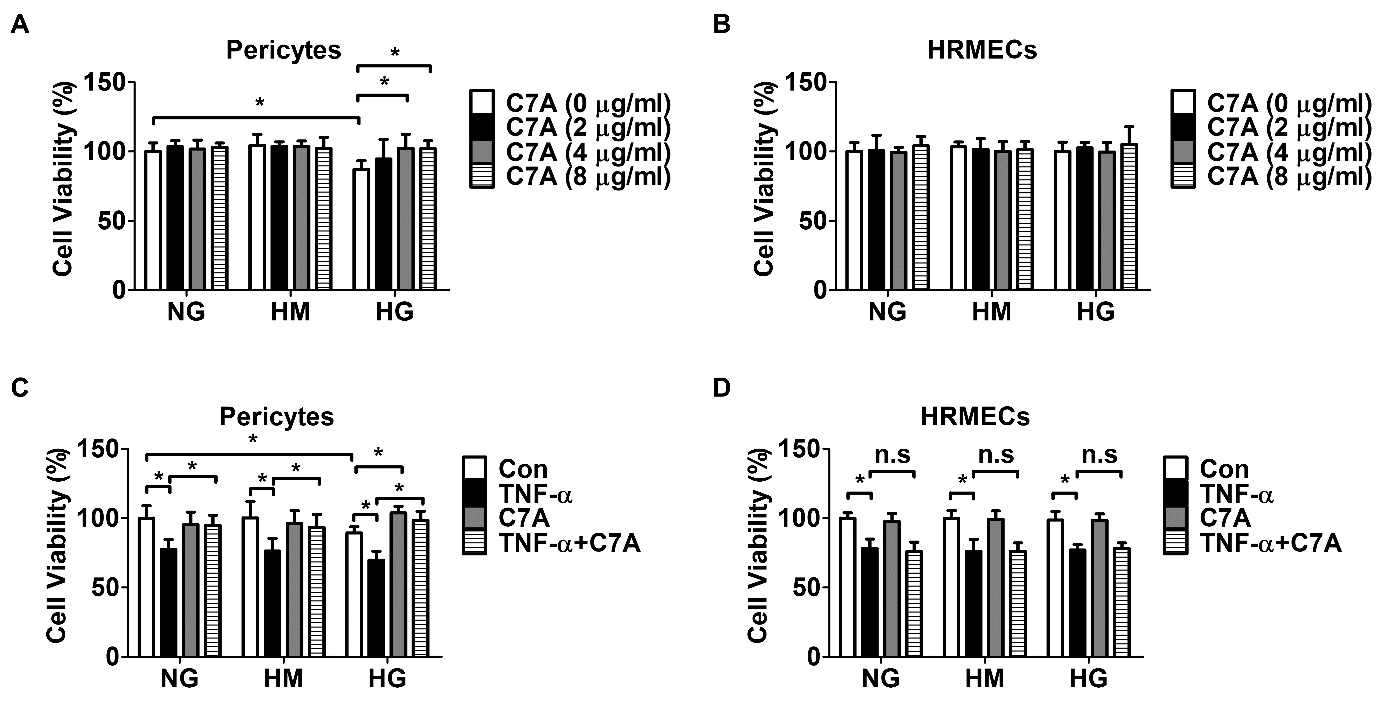
**

**Supplementary Figure 3.** Effect of the compound C7A in the *U. davidiana* on cell viability of pericytes and human retinal microvascular endothelial cells (HRMECs). Pericytes (A) and HRMECs (B) were treated with C7A for 72 h at indicated doses under the conditions of high glucose (HG; 30 mM glucose). Normal glucose (NG; 5 mM glucose) and high mannitol (HM; 5 mM glucose and 25 mM mannitol) were used as controls. The cell viability was determined by the MTT assay. The bar graph represents the means ± SD (*n* = 5). **P* < 0.05. Pericytes (C) and HRMECs (D) were treated with C7A (4 μg/ml) for 72 h under the conditions of HG, with or without tumor necrosis factor α (TNF-α) (100 ng/ml). The cell viability was determined by the MTT assay. The bar graph represents the means ± SD (*n* = 5). No significance (n.s.) indicates *P* > 0.05, **P* < 0.05.





**Supplementary Figure 4.** Effect of the compound C7A in the *U. davidiana* on survival and proliferation in pericytes and human retinal microvascular endothelial cells (HRMECs). (A-E) Pericytes and HRMECs were treated with C7A (4 μg/ml) for 72 h under the conditions of high glucose (HG; 30 mM glucose), with or without tumor necrosis factor α (TNF-α) (100 ng/ml). Normal glucose (NG; 5 mM glucose) and high mannitol (HM; 5 mM glucose and 25 mM mannitol) were used as controls. Cell apoptosis of pericytes (A) and HRMECs (B) was determined by Annexin V/PI staining and flow cytometric analysis. The apoptotic cells were expressed as a percentage of apoptotic cells in the total cell population. The bar graph represents the means ± standard deviation (SD) (*n* = 3). (C) The cleaved caspase-3 expression was determined by western blot analysis. β-tubulin were used as controls. The right histogram showed quantitative densitometric analysis. The bar graph represents the means ± standard deviation (SD) (*n* = 3). Cell proliferation of pericytes (D) and HRMECs (E) was determined by 5’-bromodeoxy-2’-uridine (BrdU) proliferation ELISA. The bar graph represents the means ± SD (*n* = 5). No significance (n.s.) indicates *P* > 0.05, **P* < 0.05.

**
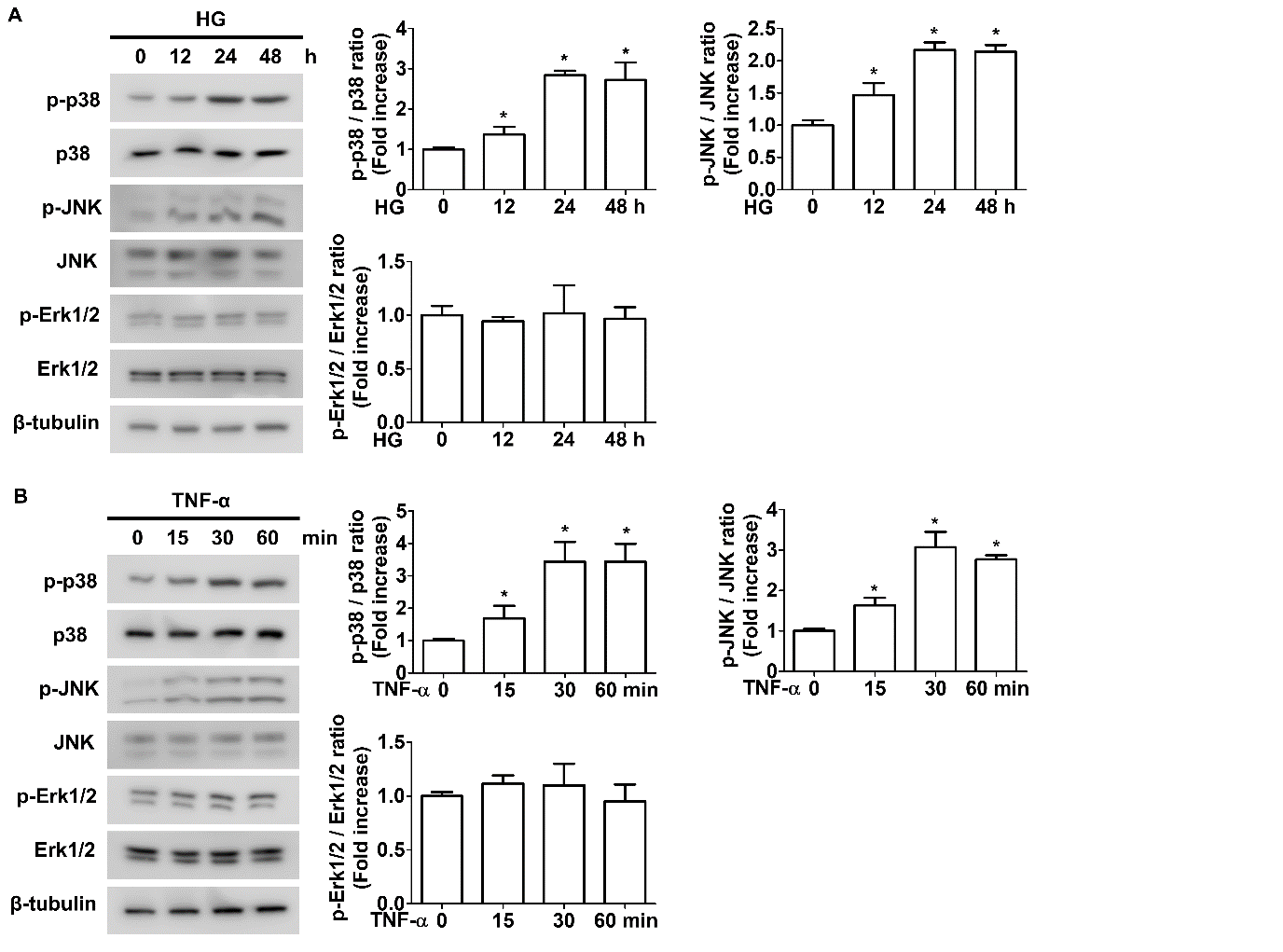
**

**Supplementary Figure 5.** Effect of high glucose and tumor necrosis factor α (TNF-α) on p38, JNK, and Erk1/2 signaling in pericytes. (A-B) Pericytes were treated with high glucose (HG, 30 mM glucose) or TNF-α (100 ng/ml) for the indicated times. The phosphorylation of p38 (p-p38), JNK (p-JNK), and Erk1/2 (p-Erk1/2) was determined by western blot analysis. p38, JNK, Erk1/2, and β-tubulin were used as controls. The right histogram showed quantitative densitometric analysis. The bar graph represents the means ± standard deviation (SD) (*n* = 3). **P* < 0.05.


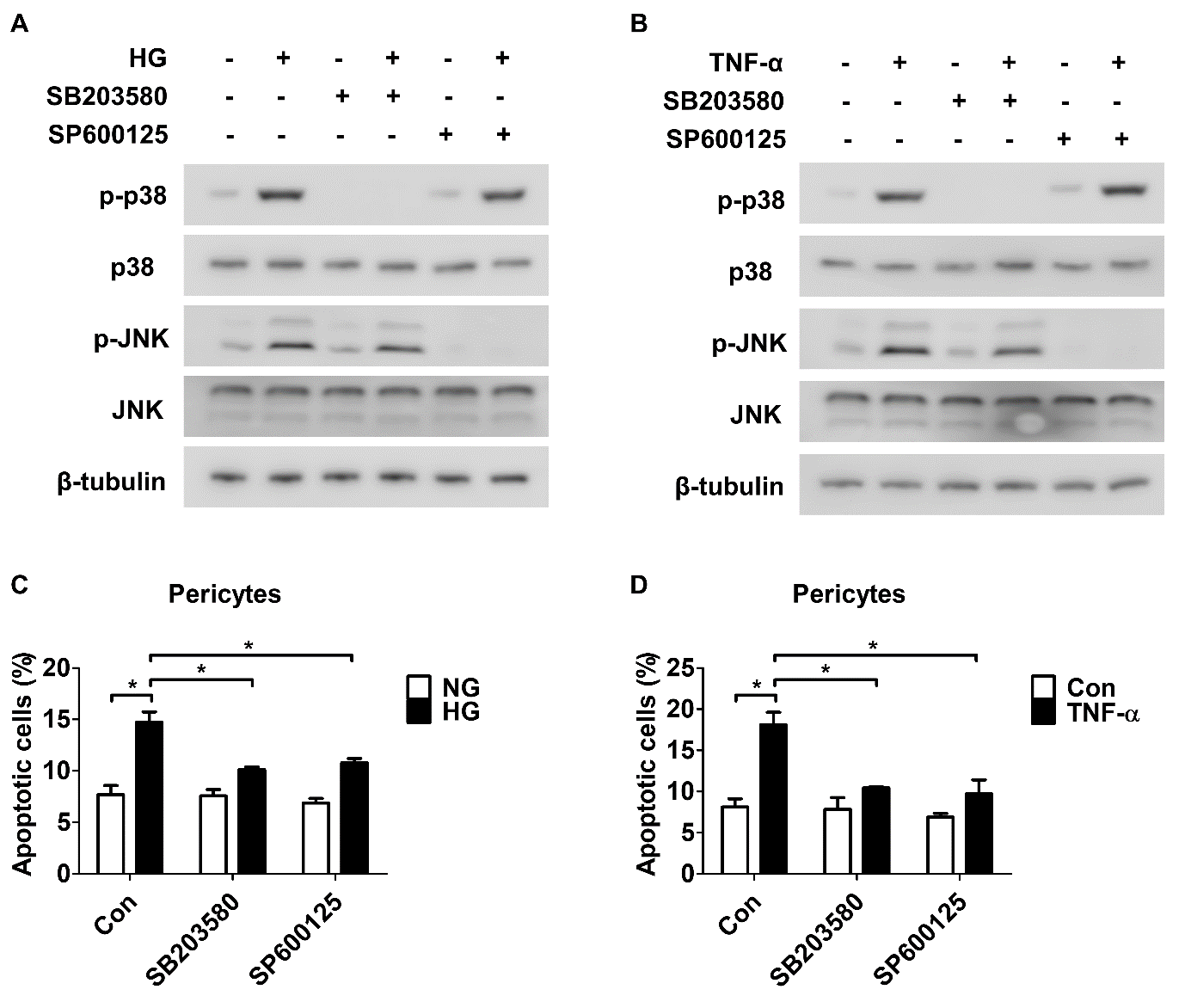


**Supplementary Figure 6.** Involvement of p38 and JNK signaling in high glucose and tumor necrosis factor α (TNF-α)-induced pericyte apoptosis. (A-B) Pericytes were treated with SB203580 (10 μM), SP600125 (10 μM), and/or TNF-α (100 ng/ml) for 30 min under conditions exposed to normal glucose (NG; 5 mM glucose) or high glucose (HG, 30 mM glucose) for 24 h. The phosphorylation of p38 (p-p38) and JNK (p-JNK) was determined by western blot analysis. p38, JNK, and β-tubulin were used as controls. (C-D) Pericytes were treated with SB203580 (10 μM), SP600125 (10 μM), and/or TNF-α (100 ng/ml) for 72 h under NG or HG conditions. Cell apoptosis was determined by Annexin V/PI staining and flow cytometric analysis. The apoptotic cells were expressed as a percentage of apoptotic cells in the total cell population. The bar graph represents the means ± standard deviation (SD) (*n* = 3). **P* < 0.05.

**
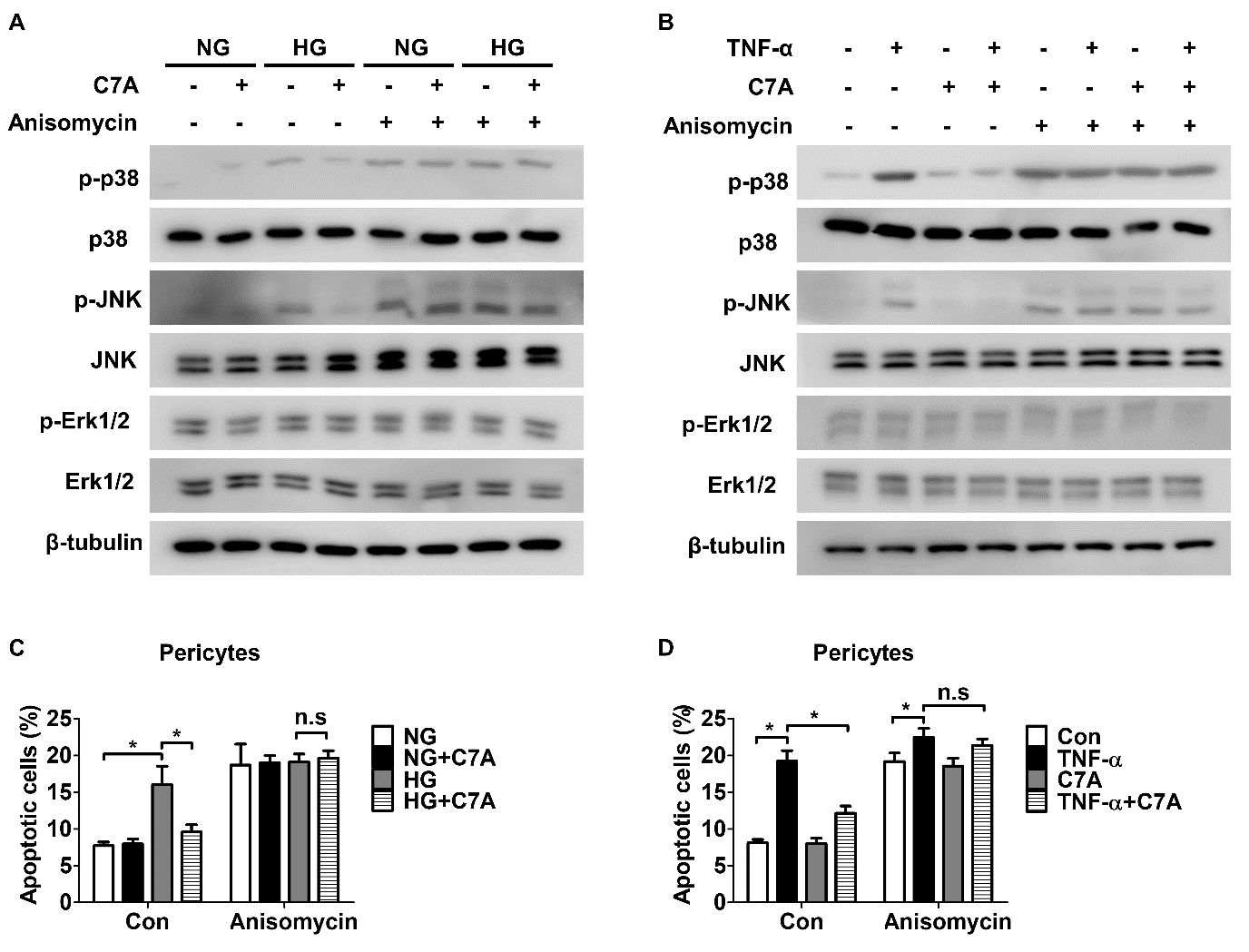
**

**Supplementary Figure 7.** Involvement of p38 and JNK signaling in the C7A-induced pericyte survival. (A-B) Pericytes were treated with C7A (4 μg/mL), tumor necrosis factor α (TNF-α) (100 ng/ml), and/or anisomycin (100 ng/ml) for 30 min under conditions exposed to normal glucose (NG; 5 mM glucose) or high glucose (HG, 30 mM glucose) for 24 h. The phosphorylation of p38 (p-p38), JNK (p-JNK), and Erk1/2 (p-Erk1/2) was determined by western blot analysis. p38, JNK, Erk1/2, and β-tubulin were used as controls. (C-D) Pericytes were treated with C7A (4 μg/mL), TNF-α (100 ng/ml), and/or anisomycin (100 ng/ml) for 72 h under NG or HG conditions. Cell apoptosis was determined by Annexin V/PI staining and flow cytometric analysis. The apoptotic cells were expressed as a percentage of apoptotic cells in the total cell population. The bar graph represents the means ± SD (*n* = 3). No significance (n.s.) indicates *P* > 0.05, **P* < 0.05.


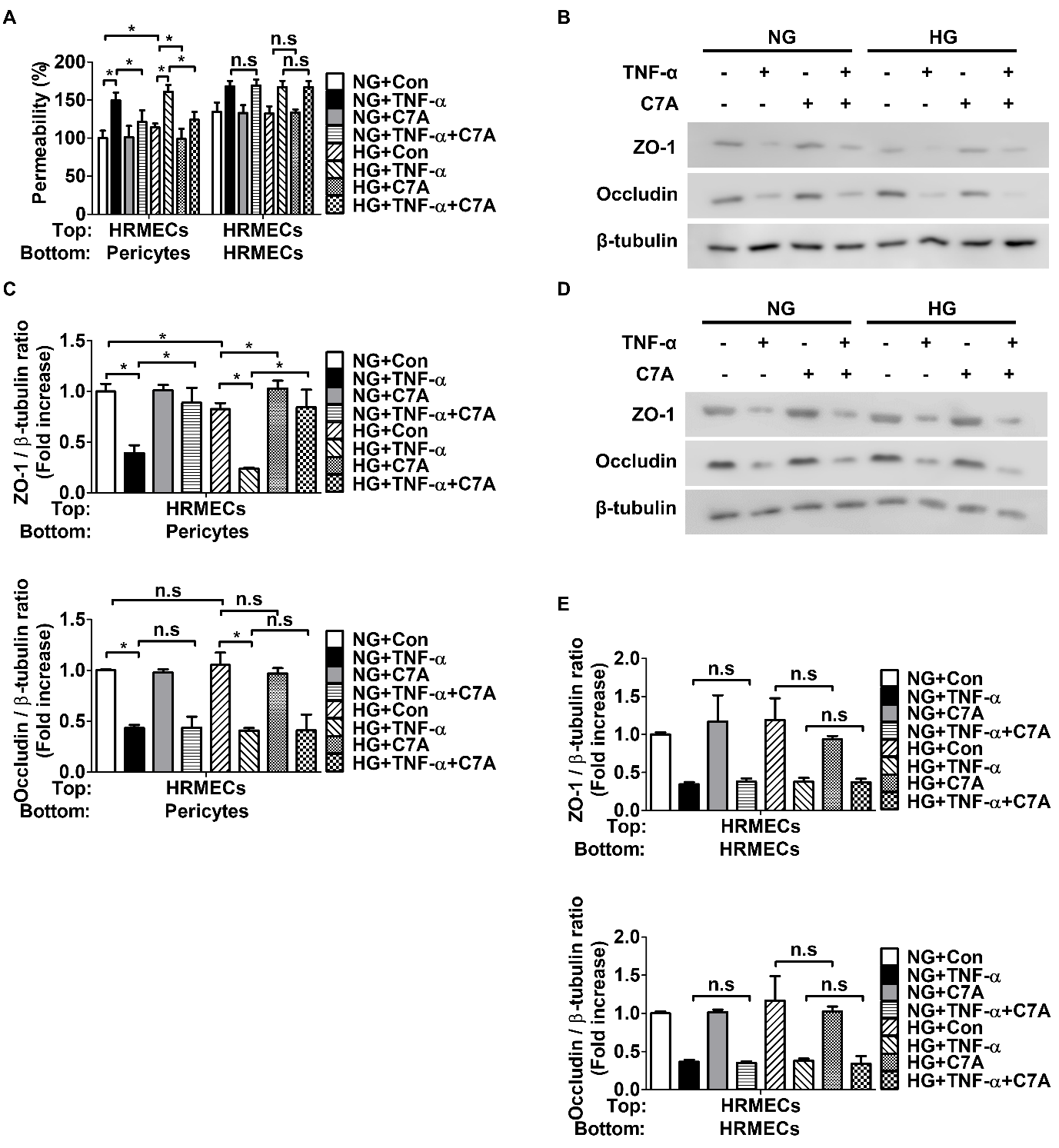


**Supplementary Figure 8.** Effect of C7A on the *in vitro* permeability in co-cultures of pericytes and human retinal microvascular endothelial cells (HRMECs) and the tight junction protein expression in HRMECs. (A) Pericytes and HRMECs were incubated on the indicated side of the Transwells and then treated with C7A (4 μg/mL) and/or TNF-α (100 ng/ml) under conditions exposed to normal glucose (NG; 5 mM glucose) or high glucose (HG, 30 mM glucose) for 72 h. The permeability was measured using Evans blue dye (*n* = 5). (B-E) The tight junction protein expression of ZO-1 and occludin was measured from the top side HRMECs lysates under conditions for co-culture of pericytes and HRMECs (B-C) or conditions for culturing only HRMECs (D-E) obtained by (A). Quantitative densitometric analysis was performed to calculate the ratio of each protein to β-tubulin (C, E). The bar graph represents the means ± SD (*n* = 3). No significance (n.s.) indicates *P* > 0.05, **P* < 0.05.
